# Supplementary material for: Association between malaria and undernutrition among pregnant women at presentation for antenatal care in health facilities in the Mount Cameroon region
Source: PLoS One. 2023 Oct 12;18(10):e0292550. doi: 10.1371/journal.pone.0292550 (PMC10569528; doi:10.1371/journal.pone.0292550)
Supplement: Attachment — Submitted filename: Reviewers comments_CA.docx [file pone.0292550.s003.docx]

**Reviewer’s Comments:**

1. Point 1: **Line 378** states, "Low maternal education levels may facilitate poor knowledge on the utilization and intake of malaria preventive measures..." This can be rephrased as "Low maternal education levels may contribute to inadequate understanding of malaria preventive measures..."
2. Point 2: Considering that the dietary diversity score was utilized to assess maternal dietary intake, it is unnecessary to include **lines 166-168,** which mention the use of 24-hour dietary recall. Both methods can be employed for dietary assessment, but only one is required.
3. Point 3: The term "undernutrition" should be used consistently, as it is sometimes referred to as inadequate dietary diversity in one context and insufficient dietary intake in another.
