# Supplementary material for: Association between malaria and undernutrition among pregnant women at presentation for antenatal care in health facilities in the Mount Cameroon region
Source: PLoS One. 2023 Oct 12;18(10):e0292550. doi: 10.1371/journal.pone.0292550 (PMC10569528; doi:10.1371/journal.pone.0292550)
Supplement: Attachment — Submitted filename: Response to Reviewers.docx [file pone.0292550.s004.docx]

Dear Editor

Many thanks for your email of 16 August, 2023 providing comments on our manuscript and inviting us to submit a second revision. We have now prepared a revised manuscript reflecting the suggestions made by the Editor and reviewers for consideration. Below we have provided a point-by-point response to the concerns and comments detailing the changes we have made in response to each point raised. The changes made to the manuscript based on the comments and concerns of the Editor and reviewers have been tracked in the revised manuscript for appraisal. We would like to thank the Editor and reviewers for taking the time to read our manuscript again and to provide thoughtful and helpful comments for revision. We believe the revised version of our manuscript represents a significant improvement on the original.

We look forward to hearing from you in due course

Vanessa Tita Jugha (on behalf of all authors)

See Authors’ responses to Editor and Reviewer’s comments in the attached document.

**Responses to Editor and Reviewers’ Concerns and Comments**

**Manuscript Title:** **Association between malaria and undernutrition among pregnant women at presentation for antenatal care in health facilities in the Mount Cameroon region**

**Authors**

Vanessa Tita Jugha^1*^, Juliana Adjem Anchang^2^, Germain Sotoing Taiwe^1^, Helen Kuokuo Kimbi^1,3,4^, Judith Kuoh Anchang-Kimbi^1^

**Version: 3**

Date: 20^th^ September, 2023

Please see authors’ responses to Editor and reviewers’ concerns and comments over leaf.

**Response to Comments and Concerns of the Editor**

**Please review your reference list to ensure that it is complete and correct. If you have cited papers that have been retracted, please include the rationale for doing so in the manuscript text, or remove these references and replace them with relevant current references. Any changes to the reference list should be mentioned in the rebuttal letter that accompanies your revised manuscript. If you need to cite a retracted article, indicate the article’s retracted status in the References list and also include a citation and full reference for the retraction notice.**

The list of references has been reviewed to ensure it is complete and up to date as requested.

**Reviewers' comments to authors and responses**

**The changes made in the revised version of our manuscript with respect to the reviewers’ comments and concerns have been highlighted in yellow, and blue in the manuscript with track changes for easy appraisal.**

**Point 1: Line 378 states, "Low maternal education levels may facilitate poor knowledge on the utilization and intake of malaria preventive measures..." This can be rephrased as "Low maternal education levels may contribute to inadequate understanding of malaria preventive measures..."**

The correction (**now line 375**) have been effected as requested. Please see track changes (highlighted in yellow) for the correction.

**Point 2: Considering that the dietary diversity score was utilized to assess maternal dietary intake, it is unnecessary to include lines 166-168, which mention the use of 24-hour dietary recall. Both methods can be employed for dietary assessment, but only one is required.**

As requested, lines 166-168 has been deleted in the revised version of our manuscript.

**Point 3: The term "undernutrition" should be used consistently, as it is sometimes referred to as inadequate dietary diversity in one context and insufficient dietary intake in another.**

The phrase ‘inadequate dietary diversity’ and ‘insufficient dietary nutrient intake’ have been edited to “undernutrition” for consistency in our revised version of the manuscript. Please see track changes (highlighted in blue) for the corrections.
